# First Report of Two *Jaculus* Rodents as Potential Reservoir Hosts of *Leishmania* Parasites in Tunisia

**DOI:** 10.3390/microorganisms10081502

**Published:** 2022-07-25

**Authors:** Wissem Ghawar, Melek Chaouch, Afif Ben Salah, Mohamed Ali Snoussi, Sadok Salem, Ghassen Kharroubi, Said Chouchen, Amor Bouaoun, Dhafer Laouini, Jihene Bettaieb, Souha Ben Abderrazak

**Affiliations:** 1 Department of Medical Epidemiology, Institut Pasteur de Tunis, Tunis 1002, Tunisia; afifbs@agu.edu.bh (A.B.S.); mohamedali.snoussi@pasteur.tn (M.A.S.); sadok-salem@live.fr (S.S.); kharroubighassen@gmail.com (G.K.); bettaiebjihene@yahoo.fr (J.B.); 2 Laboratory of Transmission, Control and Immunobiology of Infections (LR16IPT02), Institut Pasteur de Tunis, Tunis 1002, Tunisia; dhafer_l@yahoo.ca; 3 Clinical Investigation Center (CIC), Institut Pasteur de Tunis, Tunis 1002, Tunisia; chouchensaid@gmail.com (S.C.); amor.bouaoun@gmail.com (A.B.); 4 Campus Universitaire Farhat Hached, University Tunis El Manar, Tunis 1068, Tunisia; mcmelek@msn.com (M.C.); souha.benabderrazakklilib@pasteur.tn (S.B.A.); 5 Laboratory of Medical Parasitology, Biotechnology and Biomolecules (LR16IPT06), Institut Pasteur de Tunis, Tunis 1002, Tunisia; 6 Laboratory of Bioinformatics, Biomathematics and Biostatistics (LR16IPT09), Institut Pasteur de Tunis, Tunis 1002, Tunisia; 7 Faculty of Medicine of Tunis, University Tunis El Manar, Tunis 1068, Tunisia; 8 Department of Family and Community Medicine, College of Medicine and Medical Sciences (CMMS), Arabian Gulf University (AGU), Manama 329, Bahrain; 9 Health Regional Directorate of Tataouine, Administrative City, Tataouine Nouvelle, Tataouine 3263, Tunisia

**Keywords:** lesser Egyptian jerboa, African hammada jerboa, *Leishmania major*, *Leishmania tropica*, rodent reservoir host, cutaneous leishmaniasis, Tunisia

## Abstract

This study shows, for the first time, natural *Leishmania* infection among *Jaculus* spp. in an endemic region of Tataouine, South Tunisia. To better characterize the transmission cycles in this complex focus of mixed transmission, *Leishmania* detection and species identification were performed by direct examination, internal transcribed spacer-1 (ITS1)-PCR-restriction fragment length polymorphism (RFLP), and sequencing of *Jaculus* (*J*.) *jaculus* (Linnaeus, 1758) and *J*. *hirtipes* (Lichtenstein, 1823) rodent species, which are frequently encountered in this area. *Leishmania* parasites were observed in 19 (41.3%) smears, while DNA parasites were detected in 28 (60.9%) *Jaculus* spp. spleens; among them, 12 (54.5%) were from 22 *J*. *jaculus* individuals and 16 (66.7%) were from 24 *J*. *hirtipes* individuals. *Leishmania* parasites were confirmed as *Leishmania* (*L*.) *killicki* (syn. *L*. *tropica*) in two *J*. *hirtipes* individuals (4.3%) and *L*. *major* (n = 24; 52.2%) in 10 *J. jaculus* and 14 *J. hirtipes* individuals. This finding represents the first evidence of natural infection with *Leishmania* parasites in rodents belonging to the *Jaculus* genus, providing the rationale to consider them as potential reservoir hosts of Old World *Leishmania* parasites in Tunisia and North Africa.

## 1. Introduction

Leishmaniasis is caused by protozoan parasites of the genus *Leishmania*. Among the three main clinical manifestations of this disease (cutaneous, visceral, and mucocutaneous), the cutaneous form is the most abundant [1]. In Tunisia, human cutaneous leishmaniasis (CL) is widely distributed and is prevalent in central and southern Tunisia. The annual incidence of CL ranges from 1 to 10,000 cases according to climatic factors and the importance of herd immunity [2]. Furthermore, three epidemiological scenarios of cutaneous leishmaniasis have been described: sporadic (caused by *Leishmania* (*L*.) *infantum* Nicolle, 1908), chronic (caused by *L*. *tropica* Wright, 1903), and zoonotic (caused by *L*. *major* Yakimoff & Shokkor, 1914). The latter is predominant in the central and southern parts of the country [3]. In these areas, zoonotic cutaneous leishmaniasis (ZCL) circulates in an endemoepidemic mode [4] and is recognized as a serious public health problem [2,5].

Leishmaniasis caused by *L*. *major* and *L*. *infantum* are commonly zoonotic, with some species of rodents belonging to the Muridae family and domestic dogs as reservoir hosts, respectively [6,7]. In contrast, *L*. *killicki* (syn. *L*. *tropica*), the causative agent of chronic CL in urban endemic microfoci, known to be anthroponotic, has been strongly suspected to be zoonotic, especially with evidence of *Ctenodactylus* (*C*.) *gundi* (Rothmann, 1776) infection [8,9]. Several studies have shown that *Leishmania* parasites can infect small mammals in Tunisia, including the least weasel [7] and hedgehog [10]. Furthermore, the geographical distribution of CL cases caused by *L*. *killicki* in the region of Tataouine suggested the absence of the classically conventional reservoir host, *C*. *gundi*, while other small mammals and rodent species frequently encountered in the environment (Regional Directorate of Primary Care, Tataouine) could play a role as reservoir hosts, which remain to be identified. Rodent species belonging to the *Jaculus* genus were suspicious due to their wide distribution in central and southern Tunisia [11]. Moreover, these rodents were frequently faced by the regional health workers in Tataouine (Regional Directorate of Primary Care, Tataouine). This clinical and epidemiological context justified this study, which aimed to investigate rodent species belonging to the *Jaculus* genus in an old emerging focus of CL, that is, the Tataouine Governorate in southeastern Tunisia, to explore them as novel reservoir hosts of *Leishmania* parasites in the region.

This will better characterize transmission cycles and provide stronger evidence for intervention policies.

## 2. Materials and Methods

### 2.1. Rodent Trapping and Identification

Rodents were collected between May 2016 and June 2018 using butterfly nets at two study sites: BniMhira and Guermessa, belonging to the Tataouine Governorate, South Tunisia [12].

Some of the captured rodents (n = 41) were identified by amplification of the mitochondrial complete cytochrome b (*Cytb*) gene, as previously described [12].

To identify all captured rodents consistently (n = 46), we also used polymerase chain reaction (PCR) amplification of the mitochondrial partial *Cytb* gene after DNA extraction from their spleens using the QIAamp^®^ DNA Mini Kit (QIAgen, Hilden, Germany) according to the manufacturer’s instructions. Modified vertebrate-universal specific primers (cytB1-F and cytB2-R) were used to amplify a 370 bp segment [13]. PCR reactions were conducted in a 50 μL reaction volume, including 0.2 mM dNTPs, 2.5 U of Taq DNA polymerase (GE Healthcare, Chicago, IL, USA), and 60 pmol of each primer (Forward 5′-CCATCCAACATYTCADCATGATGAAA-3′ and Reverse 5′-GCHCCTCAGAATGATATTTGKCCTCA-3′). Reactions were performed in an Applied Biosystems GeneAmp PCR System 2700 (Foster City, CA, USA) using the following cycling conditions: an initial denaturation step at 94 °C for 5 min; 30 cycles of 94 °C for 1 min, 54 °C for 1 min, and 72 °C for 1 min; and a final elongation step at 72 °C for 10 min. The amplicons were electrophoresed on 1% agarose gels and visualized under UV light. PCR products were purified using a QIAquick PCR purification kit (QIAGEN, Hilden, Germany) according to the manufacturer’s instructions. Amplicon sequencing was performed using a conventional Big Dye Terminator Cycle Sequencing Ready Reaction Kit (Perkin Elmer, Applied Biosystems, Foster City, CA, USA) and an ABI373 automated DNA sequencer (Thermo Fisher Scientific, Waltham, MA, USA). The obtained sequences were edited using ChromasPro software (Technelysium Pty Ltd., South Brisbane, Australia). For homology searches, sequences were submitted to the National Center for Biotechnology Information (NCBI) server using the Basic Local Alignment Search Tool (BLAST) (http://blast.ncbi.nlm.nih.gov (accessed on 22 March 2022)). Accession numbers of the sequences are listed in Supplementary Table S1.

### 2.2. Detection of Infection by Leishmania Parasites

#### 2.2.1. Clinical Examination

Before autopsy, each rodent was examined for cutaneous lesions in different parts of the body and skin. Clinical manifestations, including hyperpigmentation of the higher edge of the ear, depilation, small nodules, infiltration, and/or partial or total organ destruction, were recorded as previously described [7].

#### 2.2.2. Parasitological Diagnosis

Extracts from two macerated ears of each caught rodent were subjected to microscopic examination (Direct Exam) and parasite culture in Novy–MacNeal–Nicolle (NNN) medium [7].

#### 2.2.3. Molecular Screening

DNA extracted from the spleen of each rodent was used for molecular diagnosis. The presence of *Leishmania* DNA was tested by PCR targeting ribosomal internal transcribed spacer 1 (ITS1) present in numerous copies of *Trypanosoma* spp., including *Leishmania* [14,15]. Amplification was performed using LITSR 5′-CTTG GATCATTTTCCGATG-3′ and L5.8S 5′-TGA TAC CAC TTA TCG CAT T-3′ primers, as previously described [15]. The WHO reference strains *L*. *major* MON-25 MHOM/TN2009/S600, *L*. *tropica* MON-8 MHOM/TN/2011/MX, and *L*. *infantum* MON-1 MHOM/TN/80/IPT1 were used as positive controls.

#### 2.2.4. Leishmania Parasite Typing

Restriction fragment length polymorphism (RFLP) analysis of ITS1-PCR products digested with *Hae*III restriction enzyme (Invitrogen, Waltham, MA, USA) was employed to identify the detected *Leishmania* spp. [15]. In fact, *L*. *major* samples present two fragments of 132-bp and 206-bp, *L*. *killicki* three fragments of 188-bp, 57-bp, and 26-bp; and *L*. *infantum* three fragments of 187-bp, 72-bp, and 55-bp [15].

ITS1-PCR products were directly purified and sequenced in both directions using a conventional Big Dye Terminator Cycle Sequencing Ready Reaction Kit (Perkin Elmer, Applied Biosystems, Foster City, CA, USA) and an ABI373 Automated DNA Sequencer (Thermo Fisher Scientific, Waltham, MA, USA).

#### 2.2.5. Collection of Publicly Available Sequences and Phylogenetic Analyses

For our study, we searched for partial and complete ITS1 sequences belonging to *Leishmania* parasites in the NCBI database using the following keywords: *Leishmania*, internal transcribed spacer 1, ITS1, Tunisia, host, and rodents. Three reference sequences for *Leishmania* spp. (accession numbers: KF152937, MK474651.1, and MK474641.1 for *L*. *major*, *L*. *killicki*, and *L*. *infantum*, respectively) were used to identify the sequences.

Moreover, two datasets were collected: the first included all sequences originating from Tunisia (Supplementary Table S2) and the second regrouped all sequences detected from rodent hosts worldwide (Supplementary Table S3). The retrieved sequences were aligned using the MEGA 6 software (Pennsylvania State University, State College, PA, USA) [16] to study the genetic variability of the ITS1 fragment from the two datasets. The output was edited and regions of unambiguously aligned sequences were retained for the final analysis. Accession numbers of sequences used in the present phylogenetic study are provided in Supplementary Tables S2 and S3.

Maximum likelihood (ML) phylogenetic analysis “SMS: Smart Model Selection in PhyML” (Montpellier University, Montpellier, France) [17] was run to determine the most suitable model of DNA evolution for the two haplotype sets of sequences using hierarchical log-likelihood-ratio tests and the Akaike information criterion.

The best evolutionary model for both datasets was the GTR +G model with estimated gamma distribution shape parameters of 0.915 and 0.497 based on the Tunisian sequences (Supplementary Table S2) and those detected among rodents (Supplementary Table S3), respectively.

Furthermore, identified haplotypes from the two sets of sequences, and their population genetic analysis, including (i) the number of haplotypes (*H*), (ii) the haplotype diversity (*H*d), (iii) the nucleotide diversity (π), (iv) the number of segregating sites (*S*), (v) the total number of mutations (*η*), (vi) the average number of nucleotide differences (*k*), and (vii) the Tajima’s *D* test for neutrality, were realized and calculated using the DnaSP (Julio Rozas & Universitat de Barcelona, Barcelona, Spain) [18].

These models were used to generate ML haplotype phylogenetic trees with PhyML 3.0 (Montpellier University, Montpellier, France) [19] available in SeaView V4 (Lyon 1 University, Lyon, France) [20], using optimization options and 1000 bootstrap replicates. 

### 2.3. Statistical Analysis

Categorical variables are summarized as frequency counts and percentages. Comparisons between group percentages were performed using the chi-squared test or Fisher’s exact test. Statistical significance was set at *p* ≤ 0.05. All statistical analyses were performed using STATA software version 11 (StataCorp LLC, TX, USA).

## 3. Results

### 3.1. Rodent Identification

A total of 46 rodents belonging to the *Jaculus* genus were captured. Phylogenetic analysis of the complete *Cytb* gene, as previously described [12], identified 21 as belonging to *Jaculus* (*J*.) *jaculus* and 20 to *J. hirtipes*. A total of 39 short *Cytb* sequences were successfully amplified, allowing the identification of all rodent populations (Supplementary Table S1). Blasting 360 bp sequences allowed us to identify all the captured rodents as 22 (47.8%) belonging to *J*. *jaculus* and 24 (52.2%) to *J. hirtipes*. No discordance was observed between the two studies. The distribution of these rodents according to capture biotope and gender is illustrated in Table 1.

### 3.2. Leishmania Infection

**Figure 1:**
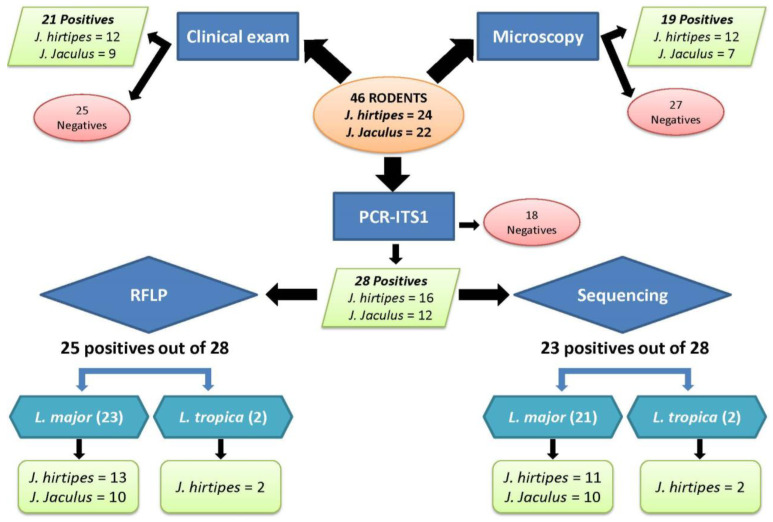
Flowchart summarizing the process and the results of *Leishmania* diagnosis and identification among *Jaculus* species. PCR-ITS1: PCR targeting ribosomal internal transcribed spacer 1; RFLP: Restriction fragment length polymorphism.

The process of *Leishmania* diagnosis and identification among *Jaculus* rodents is shown in the flowchart in Figure 1.

### 3.3. Clinical Manifestations

**Figure 2:**
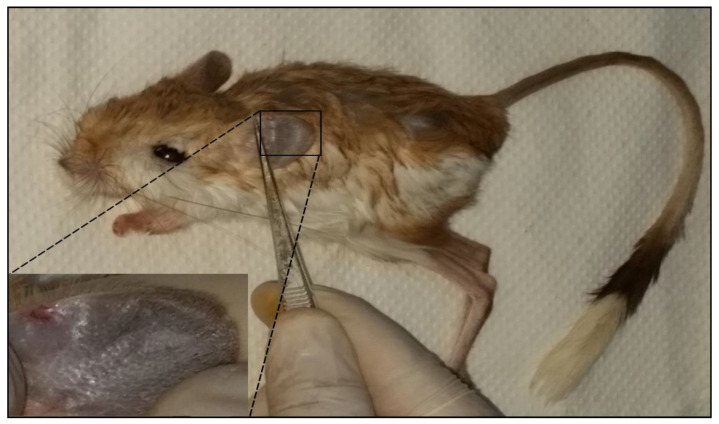
*Jaculus jaculus* presenting leishmaniasis clinical manifestation as a small nodule on the higher edge of the ear.

Clinical examination of captured rodents revealed the presence of skin lesions as small nodules in the back and tail, and, more specifically, in both ears (Figure 2) among 45.7% (21/46) of the rodents tested. Specifically, nine (40.9%) *J. jaculus* and twelve (50%) *J. hirtipes* presented at least one clinical sign compatible with CL.

### 3.4. Leishmania Infection Prevalence

The presence of a confirmed *Leishmania* infection varied among *Jaculus* specimens, according to the technique used (Figure 1).

#### 3.4.1. Parasitological Methods

**Figure 3:**
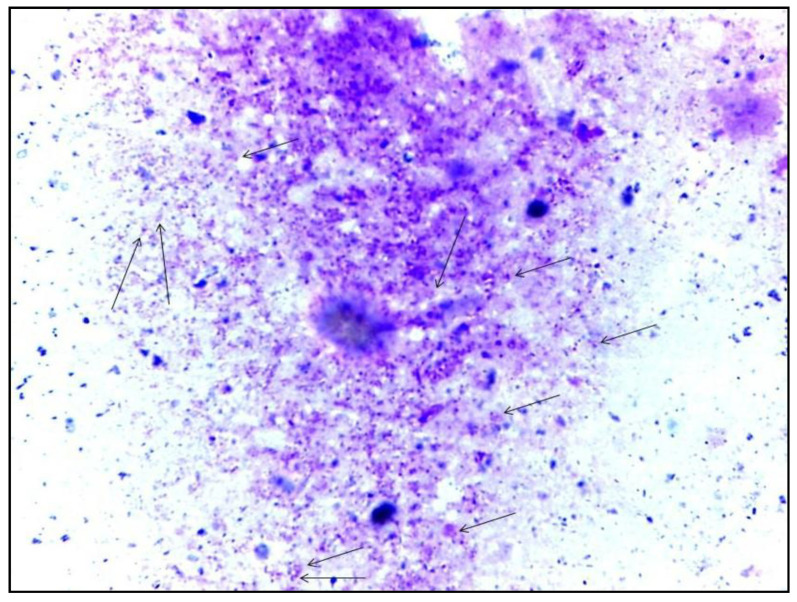
*Leishmania* amastigotes in Giemsa-stained smears from the ear of *Jaculus* species under oil immersion (×1000) (Leica DM50).

For all studied rodents, the infection prevalence was 41.3% (19/46) by microscopy (Figure 3). Specifically, seven (31.8%) *J. jaculus* and twelve (50%) *J. hirtipes* showed the presence of amastigote forms. Unfortunately, we did not succeed in growing *Leishmania* isolates from these rodents on Novy–MacNeal–Nicolle (NNN) medium because of the high rate of contamination.

#### 3.4.2. Molecular Methods

*Leishmania* infection determined by ITS1-PCR among all the studied rodents reached 60.9% (28/46) and varied according to *Jaculus* spp. (Figure 1). Specifically, 12 (54.5%) *J. jaculus* and 16 (66.7%) *J. hirtipes* samples were positive.

#### 3.4.3. Combined Methods

Combining these two methods (parasitological and molecular), *Leishmania* parasites were detected in 37 of the 46 studied *Jaculus* individuals, reaching a positivity rate of 80.4%. Moreover, there is a non-significant difference between positivity rate of both rodent species (*p*-value not significant); it was 77.3% (17/22) among *J*. *jaculus* and 83.3% (20/24) among *J*. *hirtipes*.

A comparison between the overall detected infection and clinical signs revealed a high rate of asymptomatic infection, rising to 59.5% (22/37). According to the rodent spp., asymptomatic infection was 64.7% (11/17) versus 55% (11/20) among *J*. *jaculus* and *J*. *hirtipes*, respectively (*p*-value not significant).

### 3.5. Leishmania Parasite Typing

*Leishmania* parasite typing was performed using two methods (Figure 1). First, RFLP analysis allowed the identification of 89% (25/28) of the detected positive ITS1-PCR, such as 23 *L*. *major* and 2 *L. killicki*. In fact, 10 *J*. *jaculus* (45.5%) and 13 *J*. *hirtipes* (54.2%) individuals were infected by *L*. *major*, whereas *L*. *killicki* were exclusively found in two *J*. *hirtipes* (8.3%) individuals.

**Figure 4:**
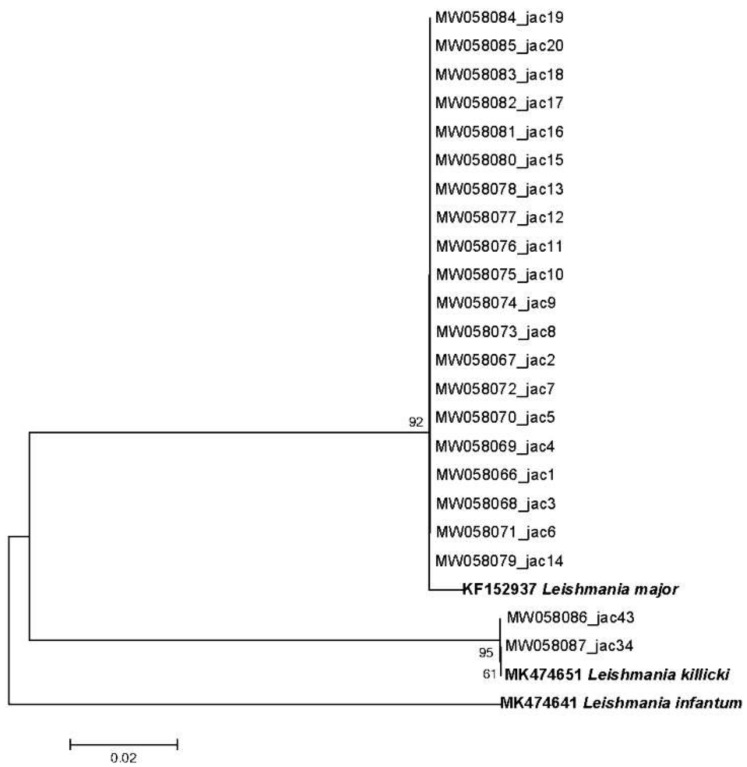
Maximum likelihood phylogenetic tree constructed with *Leishmania* ITS1 sequences detected among *Jaculus* species and three sequences used as references (bold-faced). The numbers above each sequence are their accession numbers, and those above the branches are bootstrap percentages of 1000 replications.

Second, 82% (23/28) of the ITS1-PCR-positive samples were successfully sequenced, and their ML phylogenetic tree was constructed using three reference sequences for each *Leishmania* spp. (accession numbers of *Leishmania* ITS1 sequences used as references: KF152937, MK474651.1, and MK474641.1 for *L*. *major*, *L*. *killicki,* and *L*. *infantum*), confirming the results of the RFLP analysis (Figure 4).

Only four samples were not double-checked by either method: one of them was not identified by RFLP and found to be *L*. *major* by sequencing, and three identified by RFLP as *L*. *major* were not successfully sequenced. Finally, two samples were not species-identified either by RFLP or sequencing.

Ultimately, combining the results of both methods, we identified *Leishmania* spp. in 26 samples: 10 *J*. *jaculus* (45.5%) and 14 *J*. *hirtipes* (58.3%) were found to be infected by *L*. *major* and 2 *J*. *hirtipes* (8.3%) were found to be infected by *L*. *killicki*.

### 3.6. Leishmania Phylogenetic Analysis

**Figure 5:**
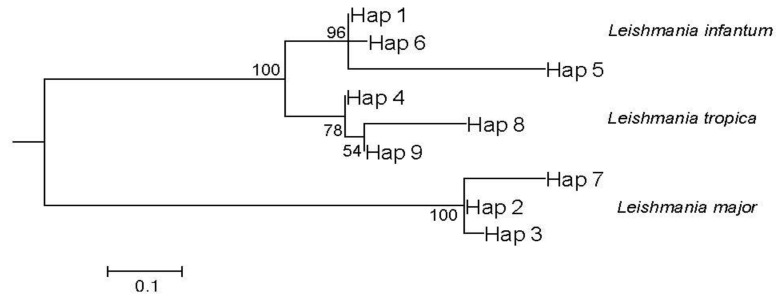
Maximum likelihood phylogenetic tree for 9 haplotypes of all Tunisian *Leishmania* parasites based on ITS1-rDNA gene sequences. Sequences fitting for each haplotype were defined in Supplementary Table S2. The numbers above the branches are bootstrap percentages of 1000 replications.

Phylogenetic analysis of all Tunisian *Leishmania* ITS1 sequences, performed using the ML method, showed three strongly supported clades with high bootstrap values (100) separating *Leishmania* parasite spp. (Figure 5).

Interestingly, among each of the three clades, we found three different haplotypes: one dominant haplotype encompassing the majority of the sequences regardless of their location, origin, and hosts, and two other haplotypes encompassing only one sequence each. The sequences detected among *Jaculus* spp. were grouped among the dominant haplotype for either *L*. *major* (Hap 2) or *L*. *tropica* (Hap 4).

Indices of genetic diversity among the haplotypes determined for the three species of *Leishmania* parasites are summarized in Table 2. Tajima’s *D* test, which was used to determine the extent of neutral selection among these Tunisian parasites, generated a positive value (*D* = 1.2243; *p* > 0.1).

**Figure 6:**
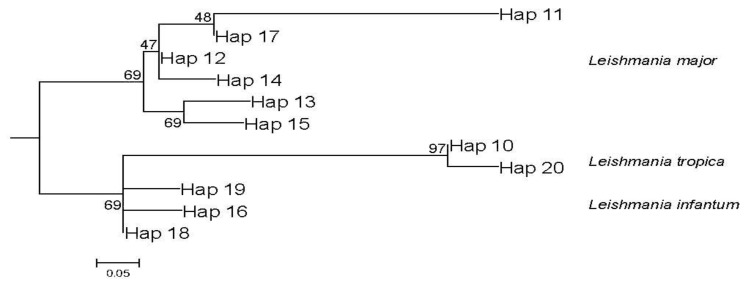
Maximum-likelihood phylogenetic tree for 11 haplotypes of all rodent hosts–*Leishmania* parasites based on ITS1-rDNA gene sequences. Sequences fitting for each haplotype are defined in Supplementary Table S3. Haplotypes are numbered from 10 to 20 to avoid confusion with the previous figure. The numbers above the branches are bootstrap percentages of 1000 replications.

The same topology was also observed in a phylogenetic study of the worldwide ITS1 *Leishmania* sequences detected in rodent reservoir hosts. These sequences were sufficient to discriminate *Leishmania* species (Figure 6).

The number of haplotypes increased compared to the previous phylogenetic analysis. In fact, *L*. *tropica* and *L*. *infantum* clades were represented by two and three haplotypes, respectively, with a dominant haplotype, while the remaining haplotypes contained a single sequence, whereas the *L*. *major* clade comprised six haplotypes with two dominant haplotypes (Hap 12 and Hap 17). Interestingly, each of the latter haplotypes comprised *L*. *major* ITS1 sequences from a single geographic location, namely Tunisia and Iran, and each of the four remaining haplotypes contained a single sequence. Similar to the previous analysis, the sequences described in this study were grouped in the dominant haplotype for both parasite spp. (Hap 17 for *L*. *major* clade and Hap 10 for *L*. *tropica* clade). The indices of genetic diversity among the haplotypes (Figure 6) are summarized in Table 2. Tajima’s *D* test, used to determine the extent of neutral selection among parasites detected from different rodent reservoir hosts, generated a negative value (*D* = −0.6; *p* > 0.05).

## 4. Discussion

To our knowledge, this is the first study to shed light on the importance of *Leishmania* infection and its clinical manifestations in two rodent spp. belonging to the *Jaculus* genus. This novel finding could constitute a breakthrough in understanding the transmission cycles of Old World cutaneous leishmaniasis, with implications for control. Confirmed or suspected reservoir hosts have been identified following the discovery of *Leishmania* parasites in numerous studies [7,9,21,22,23,24,25,26].

In fact, *Jaculus* rodents have been found to be infected with many ecto- and endoparasites [27,28,29]. Although *Jaculus* spp. were found to be susceptible to experimental infection with *Leishmania* parasites, the infection seems to be chronic and does not impair animal health [30,31], even in Tunisia with *L*. *tropica* [32]. However, despite several attempts since 1986, *Leishmania* parasites have never been isolated and/or detected in these rodents [33,34,35,36,37]. It is worth noting that all of these studies were based on a small sample size of rodents (<10) and were conducted only using parasitological diagnostic methods.

In our study, the *Leishmania* infection prevalence among *Jaculus* spp. was 41.3% and 60.9% using parasitological and molecular tools, respectively. These high infection rates fluctuate depending on the rodent spp. Indeed, they increased among *J*. *hirtipes* using both methods (50% by direct examination and 66.7% by ITS1-PCR), while they reached only 31.8% by direct examination and 54.4% by ITS1-PCR among tested *J*. *jaculus* individuals. Despite these variations, the overall infection prevalence in these rodent spp. (more than 80%) remains the highest compared to previous proportions observed among the potential and/or confirmed rodent reservoir hosts in Tunisia using parasitological, serological, or molecular tools [7,9,21,22,23,38].

Approximately 60% of the overall infections among these rodents were asymptomatic, similar to previous findings in rodent reservoir hosts [9,39]. This might be explained by the fact that *Leishmania* parasites can disseminate to the spleen during the early stages of infection in asymptomatic animals [40]. This finding, in addition to the clinical manifestation and location of lesions, is in agreement with previous studies performed on reservoir hosts in Tunisia [7,9,22]. This could indicate a long history of co-evolution between these rodent spp. and the detected *Leishmania* parasites.

RFLP and sequencing approaches, followed by the construction of a phylogenetic tree (Figure 4), allowed for the identification of *Leishmania* spp. infecting these rodents. Surprisingly, both rodent *Jaculus* genera were found to be infected with *L*. *major* parasites. However, only *J*. *hirtipes* was found to be infected with *L*. *killicki*. Moreover, two specimens of *J*. *hirtipes* infected with *L*. *killicki* were captured in Guermessa, which is known to be an old focus of CL owing to *L*. *killicki* in Tunisia (according to the Regional Health Directorate and National Control Program of Cutaneous Leishmaniasis). This concomitant infection with two different *Leishmania* spp. has already been described in other rodents, such as *Nesokia indica* (*L*. *major* and *L*. *infantum*) [41], *Psammomys* spp. (*L*. *major*, *L*. *killicki*, and *L*. *infantum*) [21]; *C*. *gundi* (*L*. *major* and *L*. *killicki*), ref. [9] and *Mus musculus* Linnaeus, 1758 (*L*. *tropica* and *L*. *infantum*) [25].

Similar to other rodent reservoir host studies conducted worldwide, our phylogenetic study revealed a low number of available *Leishmania* ITS1 sequences, regardless of the origin of the host in Tunisia (≤50 sequences (Supplementary Tables S2 and S3)). Furthermore, we confirmed that ITS1 sequences could segregate between *Leishmania* spp., even for small samples [9,42,43,44,45,46,47,48,49,50].

The Tunisian *Leishmania* phylogenetic tree (Figure 5) allowing the separation of the parasite spp. failed to show clear grouping according to the sequences, geographical origin, or related hosts. Moreover, this result confirms the heterogeneity of circulating *Leishmania* parasites in the country, with a dominant haplotype for each parasite sp., even though two more haplotypes were detected containing a single sequence for each *Leishmania* spp. (Supplementary Table S2).

Furthermore, the genetic diversity among Tunisian sequences was high, as evidenced by the high haplotype diversity (*H*d = 0.707) and nucleotide diversity (π = 0.15), which were comparable to the values determined from kinetoplast DNA sequences of other tested *Leishmania* complexes [51]. This heterogeneity has been reported using other genetic markers among Tunisian *Leishmania* spp. in previous studies [52,53,54,55].

In addition, the positive value of Tajima’s *D* test suggests a recent population bottleneck or balancing selection [51,56,57]. The latter seems to be more realistic and can explain the *Leishmania* parasite evolution in Tunisia, despite the fact that this value was not significant, most likely because of the small number of available *Leishmania* ITS1 sequences. In addition, genetic variability among *Leishmania* ITS1 genes at the species level, although not clearly established due to the reduced number of sequences of the determined haplotypes (one sequence for some haplotypes), can be related to the zymodemes of *Leishmania* parasites circulating in the country. As established for Tunisia, *L*. *major* MON-25 and *L*. *killicki* MON-8 were the exclusive zymodemes for these dermotropic species, whereas the viscerotropic species were represented by three different zymodemes: MON-24 (the most frequent), MON-1, and MON-80 [2]. In the last decade, many other zymodemes have been detected in Tunisia (*L*. *tropica* MON-317) [55] and neighboring countries [2]. This description can explain our results and confirm the presence of other potential zymodemes. These hypotheses should be addressed in future studies.

On the other hand, *Leishmania* from the phylogenetic tree of rodent hosts (Figure 6) showed a high level of diversity, with 11 haplotypes detected, and a clear clustering according to the geographical origin in *L. major* clade (Supplementary Table S3). Similarly, the genetic diversity among rodent host sequences was high, as revealed by the high haplotype diversity (*H*d = 0.765) and nucleotide diversity (π = 0.03).

In addition, the negative value of the Tajima’s *D* test suggests, by definition, a population expansion or purifying selection [51]. This could explain the *Leishmania* parasite evolution among their rodent reservoir hosts, suggesting a purifying geographical link. Enriching databases with more *Leishmania* ITS1 sequences harbored by rodent reservoir hosts from all over the world could confirm this finding.

This is the first description of *Leishmania* infection in rodents belonging to the Dipodidae family. In fact, previous studies using ITS1 sequences have described *Leishmania* infection among rodents belonging to two families: seven species belonging to five genera in the Muridea family and only one species from the Ctenodactylidea family (Figure 6). Confirming that these new rodents are reservoir hosts for cutaneous leishmaniasis might pave the way for more targeted control measures.

## 5. Conclusions

The present study suggests the role of the two *Jaculus* spp. as potential reservoir hosts in the transmission cycles of *L*. *major* and *L*. *killicki* for both the former and only for *J*. *hirtipes* in the latter. Despite the small size of this study, the high proportion of infected specimens, the high density of these rodents, and their wide geographic spread in Tunisia [58] and the Old World [59] demand deeper investigations on larger sample sizes with potential striking consequences for CL transmission and control.

## Figures and Tables

**Table 1:** Distribution of the captured rodents by location and gender.

	*Jaculus* (*J*.) *jaculus* *n* = 22	*J. hirtipes* *n* = 24
Location of capture * n (%)		
− Guermessa	8 (36.0)	8 (33.3)
− BniMhira	14 (64.0)	16 (66.7)
Gender * n (%)		
− Males	4 (18.2)	6 (25.0)
− Females	18 (81.8)	18 (75.0)

*: no significant association.

**Table 2:** Indices of genetic diversity for the two studied sets of *Leishmania* sequences.

Sets of Sequences	*NS*	*H*	*S*	*η*	*k*	*H*d (SD)	π (SD)
Different hosts (Tunisia)	73	9	40	44	12.44	0.707 (0.029)	0.1575 (0.004)
Rodents hosts (all world)	71	11	16	18	2.97	0.765 (0.031)	0.0327 (0.003)

*NS*: number of sequences, *H*: number of haplotypes, *S*: number of segregating sites, *η*: total number of mutations, *k*: average number of nucleotide differences, *H*d: haplotype diversity, π: nucleotide diversity, SD: standard deviation.

## Data Availability

Cytb and ITS1 sequences were submitted to the GenBank database under accession numbers MT340265-MT340304 and MW058066-MW058088, respectively. The dresses are as follows: www.ncbi.nlm.nih.gov/genbank (accessed on 20 May 2022).

## Supplementary Materials

The following supporting information can be downloaded at: https://www.mdpi.com/article/10.3390/microorganisms10081502/s1, Table S1: Accession numbers of short and long sequences of Cytochrome b used to identify rodents as belonging to *J*. *jaculus* and *J*. *hirtipes*.; Table S2: Accessions number of Tunisian *Leishmania* sequences, their hosts, locations and haplotypes.; Table S3: Accessions number of *Leishmania* sequences detected from rodent all over the world, their hosts, locations and haplotypes.

## References

[1] Klaus S.N., Frankenburg S., Ingber A. (1999). Epidemiology of cutaneous leishmaniasis. Clin. Dermatol..

[2] Aoun K., Bouratbine A. (2014). Cutaneous leishmaniasis in North Africa: A review. Parasite.

[3] Aoun K., Amri F., Chouihi E., Haouas N., Bedoui K., Benikhlef R., Ghrab J., Babba H., Chahed M.K., Harrat Z. (2008). Epidemiology of *Leishmania* (*L*.) *infantum*, *L. major* and *L. killicki* in Tunisia: Results and analysis of the identification of 226 human and canine isolates. Bull. Soc. Pathol. Exot..

[4] Masmoudi A., Ayadi N., Boudaya S., Meziou T.J., Mseddi M., Marrekchi S., Bouassida S., Turki H., Zahaf A. (2007). Clinical polymorphism of cutaneous leishmaniasis in centre and south of Tunisia. Bull. Soc. Pathol. Exot..

[5] Ben Ismail R., Gradoni L., Gramiccia M., Bettini S., Ben Rachid M.S., Garraoui A. (1986). Epidemic cutaneous leishmaniasis in Tunisia: Biochemical characterization of parasites. Trans. R. Soc. Trop. Med. Hyg..

[6] Benikhlef R., Harrat Z., Toudjine M., Djerbouh A., Bendali-Braham S., Belkaid M. (2004). Detection of *Leishmania infantum* MON-24 in the dog. Med. Trop..

[7] Ghawar W., Snoussi M.A., Hamida N.B., Boukthir A., Yazidi R., Chaabane S., Chemkhi J., Zaatour A., Salah A.B. (2011). First report of natural infection of least weasel (*Mustela nivalis* Linnaeus, 1776) with *Leishmania major* in Tunisia. Vector Borne Zoonotic Dis..

[8] Bousslimi N., Ben-Ayed S., Ben-Abda I., Aoun K., Bouratbine A. (2012). Natural infection of North African gundi (*Ctenodactylus gundi*) by *Leishmania tropica* in the focus of cutaneous leishmaniasis, Southeast Tunisia. Am. J. Trop. Med. Hyg..

[9] Ghawar W., Bettaieb J., Salem S., Snoussi M.A., Jaouadi K., Yazidi R., Ben-Salah A. (2018). Natural infection of *Ctenodactylus gundi* by *Leishmania major* in Tunisia. Acta Trop..

[10] Chemkhi J., Souguir H., Ali I.B., Driss M., Guizani I., Guerbouj S. (2015). Natural infection of Algerian hedgehog, *Atelerix algirus* (Lereboullet 1842) with *Leishmania* parasites in Tunisia. Acta Trop..

[11] Gharaibeh B.M. (1997). Systematics, Distribution, and Zoogeography of Mammals of Tunisia.

[12] Ghawar W., Chaouch M., Ben Abderrazak S., Snoussi M.A., Salem S., Chouchen S., Bouaoun A., Ben Salah A., Bettaieb J. (2022). Evaluation of the Taxonomic Status of Lesser Egyptian Jerboa, *Jaculus jaculus*: First Description of New Phylogroups in Tunisia. Animals.

[13] Svobodova M., Alten B., Zidkova L., Dvorak V., Hlavackova J., Myskova J., Seblova V., Kasap O.E., Belen A., Votypka J. (2009). Cutaneous leishmaniasis caused by *Leishmania infantum* transmitted by *Phlebotomus tobbi*. Int. J. Parasit..

[14] Njiru Z.K., Constantine C.C., Guya S., Crowther J., Kiragu J.M., Thompson R.C., Davila A.M. (2005). The use of ITS1 rDNA PCR in detecting pathogenic African trypanosomes. Parasitol. Res..

[15] Schonian G., Nasereddin A., Dinse N., Schweynoch C., Schallig H.D., Presber W., Jaffe C.L. (2003). PCR diagnosis and characterization of *Leishmania* in local and imported clinical samples. Diagn. Microbiol. Infect. Dis..

[16] Tamura K., Stecher G., Peterson D., Filipski A., Kumar S. (2013). MEGA6: Molecular Evolutionary Genetics Analysis version 6.0. Mol. Biol. Evol..

[17] Lefort V., Longueville J.E., Gascuel O. (2017). SMS: Smart Model Selection in PhyML. Mol. Biol. Evol..

[18] Librado P., Rozas J. (2009). DnaSP v5: A software for comprehensive analysis of DNA polymorphism data. Bioinformatics.

[19] Guindon S., Dufayard J.F., Lefort V., Anisimova M., Hordijk W., Gascuel O. (2010). New algorithms and methods to estimate maximum-likelihood phylogenies: Assessing the performance of PhyML 3.0. Syst. Biol..

[20] Gouy M., Guindon S., Gascuel O. (2010). SeaView version 4: A multiplatform graphical user interface for sequence alignment and phylogenetic tree building. Mol. Biol. Evol..

[21] Ben Othman S., Ghawar W., Chaouch M., Ayari C., Chemkhi J., Cancino-Faure B., Tomas-Perez M., Alcover M.M., Riera C., Ben Salah A. (2018). First detection of *Leishmania* DNA in *Psammomys obesus* and *Psammomys vexillaris*: Their potential involvement in the epidemiology of leishmaniasis in Tunisia. Infect. Genet. Evol..

[22] Ben-Ismail R., Ben Rachid M.S., Gradoni L., Gramiccia M., Helal H., Bach-Hamba D. (1987). Zoonotic cutaneous leishmaniasis in Tunisia: Study of the disease reservoir in the Douara area. Ann. Soc. Belg. Med. Trop..

[23] Ben-Ismail R., Khaled S., Makni S., Ben Rachid M.S. (1989). Anti-leishmanial antibodies during natural infection of *Psammomys obesus* and *Meriones shawi* (Rodentia, Gerbillinae) by *Leishmania major*. Ann. Soc. Belg. Med. Trop..

[24] Davami M.H., Motazedian M.H., Kalantari M., Asgari Q., Mohammadpour I., Sotoodeh-Jahromi A., Solhjoo K., Pourahmad M. (2014). Molecular Survey on Detection of *Leishmania* Infection in Rodent Reservoirs in Jahrom District, Southern Iran. J. Arthropod. Borne Dis..

[25] Echchakery M., Chicharro C., Boussaa S., Nieto J., Carrillo E., Sheila O., Moreno J., Boumezzough A. (2017). Molecular detection of *Leishmania infantum* and *Leishmania tropica* in rodent species from endemic cutaneous leishmaniasis areas in Morocco. Parasit. Vectors.

[26] Parvizi P., Moradi G., Akbari G., Farahmand M., Ready P.D., Piazak N., Assmar M., Amirkhani A. (2008). PCR detection and sequencing of parasite ITS-rDNA gene from reservoirs host of zoonotic cutaneous leishmaniasis in central Iran. Parasitol. Res..

[27] Faleh A.B., Annabi A., López S., Said K., Ribas A. (2012). On the helminth parasites of the genus *Jaculus* (Rodentia: Dipodidae) in Tunisia: A preliminary survey study. Leb. Sci. J..

[28] Hoogstraal H. (1961). The life cycle and incidence of Hepatozoon balfouri (Laveran, 1905) in Egyptian jerboas (*Jaculus* spp.) and mites (*Haemolaelaps aegyptius* Keegan, 1956). J. Protozool..

[29] Morsy T.A., el Bahrawy A.A., al Dakhil M.M., Abdel Mawla M.M. (1994). *Babesia* infection in rodents trapped in Riyadh Region, Saudi Arabia, with a general discussion. J. Egypt. Soc. Parasitol..

[30] Al-Taqi M., Mohammed A.H. (1981). Susceptibility of Kuwaiti rodents and experimental mice to isolates of *Leishmania* spp.. Trans. R. Soc. Trop. Med. Hyg..

[31] Archibald R. (1914). A Preliminary Report on some Further Investigations on Kala Azar in the Sudan. BMJ Mil. Health.

[32] Dedet P., Ben Osman F. (1973). Sensibilite experimentale de divers rongeurs de Tunisie vis-a-vis de souches autochtones de *Leishmania tropica* (Wright, 1903) et de *Leishmania donovani* (Laveran & Mesnil, 1903). Arch. Inst. Pasteur Tunis.

[33] Bin D.S., Mostafa O.M., Abdoon A., Al-Quraishy S.A., Alqahtani A.A. (2010). Isoenzyme electrophetic characterization of *Leishmania major*, the causative agent of zoonotic cutaneous Leishmaniasis in North and West Saudi Arabia. J. Egypt. Soc. Parasitol..

[34] Hamadto H.A., Al F.A., Farrag A.B., Abdel Maksoud M.K., Morsy T.A. (2007). Zoonotic cutaneous leishmaniasis: Reservoir host and insect vector in north Sinai, Egypt. J. Egypt. Soc. Parasitol..

[35] Morsy T.A., Naser A.M., el Gibali M.R., Anwar A.M., el Said A.M. (1995). Studies on zoonotic cutaneous leishmaniasis among a group of temporary workers in North Sinai Governorate, Egypt. J. Egypt. Soc. Parasitol..

[36] Nadim A., Faghih M. (1968). The epidemiology of cutaneous leishmaniasis in the Isfahan province of Iran. I. The reservoir. II. The human disease. Trans. R. Soc. Trop. Med. Hyg..

[37] Rioux J.A., Dereure J., Khiami A., Pratlong F., Sirdar K., Lambert M. (1990). Ecoepidemiology of leishmaniasis in Syria. 1. *Leishmania major* Yakimoff and Schokhor (Kinetoplastida-Trypanosomatidae) infestation of *Psammomys obesus* Cretzschmar (Rodentia-Gerbillidae). Ann. Parasitol. Hum. Comp..

[38] Fichet-Calvet E., Jomaa I., Ben Ismail R., Ashford R.W. (2003). *Leishmania major* infection in the fat sand rat *Psammomys obesus* in Tunisia: Interaction of host and parasite populations. Ann. Trop. Med. Parasitol..

[39] Ghawar W., Toumi A., Snoussi M.A., Chlif S., Zaatour A., Boukthir A., Hamida N.B., Chemkhi J., Diouani M.F., Ben-Salah A. (2011). *Leishmania major* infection among *Psammomys obesus* and *Meriones shawi*: Reservoirs of zoonotic cutaneous leishmaniasis in Sidi Bouzid (central Tunisia). Vector Borne Zoonotic Dis..

[40] Schilling S., Glaichenhaus N. (2001). T cells that react to the immunodominant *Leishmania major* LACK antigen prevent early dissemination of the parasite in susceptible BALB/c mice. Infect. Immun..

[41] Pourmohammadi B., Mohammadi-Azni S., Kalantari M. (2017). Natural infection of *Nesokia indica* with *Leishmania major* and *Leishmania infantum* parasites in Damghan city, Northern Iran. Acta Trop..

[42] Ajaoud M., Es-sette N., Hamdi S., El-Idrissi A.L., Riyad M., Lemrani M. (2013). Detection and molecular typing of *Leishmania tropica* from *Phlebotomus sergenti* and lesions of cutaneous leishmaniasis in an emerging focus of Morocco. Parasit. Vectors.

[43] Chicharro C., Llanes-Acevedo I.P., Garcia E., Nieto J., Moreno J., Cruz I. (2013). Molecular typing of *Leishmania infantum* isolates from a leishmaniasis outbreak in Madrid, Spain, 2009 to 2012. Eur. Surveill.

[44] Ghatee M.A., Sharifi I., Kuhls K., Kanannejad Z., Harandi M.F., de Almeida M.E., Hatam G., Mirhendi H. (2014). Heterogeneity of the internal transcribed spacer region in *Leishmania tropica* isolates from southern Iran. Exp. Parasitol..

[45] Hajjaran H., Mohebali M., Mamishi S., Vasigheh F., Oshaghi M.A., Naddaf S.R., Teimouri A., Edrissian G.H., Zarei Z. (2013). Molecular identification and polymorphism determination of cutaneous and visceral leishmaniasis agents isolated from human and animal hosts in Iran. Biomed. Res. Int..

[46] Jafari R., Najafzadeh N., Sedaghat M.M., Parvizi P. (2013). Molecular characterization of sandflies and *Leishmania* detection in main vector of zoonotic cutaneous leishmaniasis in Abarkouh district of Yazd province, Iran. Asian Pac. J. Trop. Med..

[47] Mahdy M.A.K., Al-Mekhlafi H.M., Al-Mekhlafi A.M., Lim Y.A.L., Bin Shuaib N.O.M., Azazy A.A., Mahmud R. (2010). Molecular Characterization of Leishmania Species Isolated from Cutaneous Leishmaniasis in Yemen. PLoS ONE.

[48] Salloum T., Khalifeh I., Tokajian S. (2016). Detection, molecular typing and phylogenetic analysis of *Leishmania* isolated from cases of leishmaniasis among Syrian refugees in Lebanon. Parasite Epidemiol. Control.

[49] Spotin A., Rouhani S., Parvizi P. (2014). The associations of *Leishmania major* and *Leishmania tropica* aspects by focusing their morphological and molecular features on clinical appearances in Khuzestan Province, Iran. Biomed. Res. Int.

[50] Zahirnia A.H., Bordbar A., Ebrahimi S., Spotin A., Mohammadi S., Ghafari S.M., Ahmadvand S., Jabbari N., Esmaeili Rastaghi A.R., Parvizi P. (2018). Predominance of *Leishmania major* and rare occurrence of *Leishmania tropica* with haplotype variability at the center of Iran. Braz. J. Infect. Dis..

[51] Kariyawasam U.L., Selvapandiyan A., Rai K., Wani T.H., Ahuja K., Beg M.A., Premathilake H.U., Bhattarai N.R., Siriwardena Y.D., Zhong D.B. (2017). Genetic diversity of *Leishmania donovani* that causes cutaneous leishmaniasis in Sri Lanka: A cross sectional study with regional comparisons. BMC Infect. Dis.

[52] Attia H., Sghaier R.M., Gelanew T., Bali A., Schweynoch C., Guerfali F.Z., Mkannez G., Chlif S., Belhaj-Hamida N., Dellagi K. (2016). Genetic micro-heterogeneity of *Leishmania major* in emerging foci of zoonotic cutaneous leishmaniasis in Tunisia. Infect. Genet. Evol..

[53] Harrabi M., Bettaieb J., Ghawar W., Toumi A., Zaatour A., Yazidi R., Chaabane S., Chalghaf B., Hide M., Banuls A.L. (2015). Spatio-temporal Genetic Structuring of *Leishmania major* in Tunisia by Microsatellite Analysis. PLoS Negl. Trop. Dis..

[54] Chaouch M., Fathallah-Mili A., Driss M., Lahmadi R., Ayari C., Guizani I., Ben Said M., BenAbderrazak S. (2013). Identification of Tunisian *Leishmania* spp. by PCR amplification of cysteine proteinase B (cpb) genes and phylogenetic analysis. Acta Trop..

[55] Chaara D., Ravel C., Banuls A., Haouas N., Lami P., Talignani L., El Baidouri F., Jaouadi K., Harrat Z., Dedet J.P. (2015). Evolutionary history of *Leishmania killicki* (synonymous *Leishmania tropica*) and taxonomic implications. Parasit. Vectors.

[56] Shafiei R., Kalantari M., Yousefi M., Aspatwar A., Arzamani K., Bozorgomid A., Mirahmadi H., Soleimani A., Raeghi S. (2021). Bionomics and phylo-molecular analysis of *Leishmania* species isolated from human lesions using ITS1 genes in northeast of Iran. J. Parasit. Dis..

[57] El Hamouchi A., Ajaoud M., Arroub H., Charrel R., Lemrani M. (2019). Genetic diversity of *Leishmania tropica* in Morocco: Does the dominance of one haplotype signify its fitness in both predominantly anthropophilic *Phlebotomus sergenti* and human beings?. Transbound. Emerg. Dis..

[58] Ben Faleh A., Granjon L., Tatard C., Boratynski Z., Cosson J.F., Said K. (2012). Phylogeography of two cryptic species of African desert jerboas (Dipodidae: *Jaculus*). Biol. J. Linn. Soc..

[59] Moutinho A.F., Seren N., Pauperio J., Silva T.L., Martinez-Freiria F., Sotelo G., Faria R., Mappes T., Alves P.C., Brito J.C. (2020). Evolutionary history of two cryptic species of northern African jerboas. BMC Evol. Biol..

