# Supplementary material for: First Report of Two Jaculus Rodents as Potential Reservoir Hosts of Leishmania Parasites in Tunisia
Source: Microorganisms. 2022 Jul 25;10(8):1502. doi: 10.3390/microorganisms10081502 (PMC9332729; doi:10.3390/microorganisms10081502)
Supplement: Supplementary file 1 [file microorganisms-10-01502-s001.zip › microorganisms-1796274-supplementary.pdf]

Supplementary Materials

Supplementary Table S1: Accession numbers of short and long sequences of Cytochrome b used to identify rodents as belonging to *J. jaculus* and *J. hirtipes*.

Code	Long *Cytb* Sequences [1]	Short *Cytb* Sequences	Rodent Species
Jac 1	OL898641	MT340265	*J. jaculus*
Jac 2	OL898644	MT340273	*J. jaculus*
Jac 3	OL898645	MT340282	*J. jaculus*
Jac 4	OL898613	MT340292	*J. hirtipes*
Jac 5	OL898628	MT340300	*J. hirtipes*
Jac 6	OL898642	MT340301	*J. jaculus*
Jac 7	OL898626	MT340302	*J. hirtipes*
Jac 8	OL898627	MT340303	*J. hirtipes*
Jac 9	OL898624	MT340304	*J. hirtipes*
Jac 10	OL898634	MT340266	*J. jaculus*
Jac 11	OL898616	/	*J. hirtipes*
Jac 12	OL898643	MT340267	*J. jaculus*
Jac 13	OL898625	/	*J. hirtipes*
Jac 14	OL898621	/	*J. hirtipes*
Jac 15	OL898648	MT340268	*J. jaculus*
Jac 16	OL898647	MT340269	*J. jaculus*
Jac 17	OL898617	MT340270	*J. hirtipes*
Jac 18	/	MT340271	*J. hirtipes*
Jac 19	OL898646	MT340272	*J. jaculus*
Jac 20	OL898631	MT340274	*J. jaculus*
Jac 21	OL898632	MT340275	*J. jaculus*
Jac 22	/	MT340276	*J. hirtipes*
Jac 23	OL898615	MT340277	*J. hirtipes*
Jac 24	/	MT340278	*J. hirtipes*
Jac 25	OL898619	/	*J. hirtipes*
Jac 26	/	MT340279	*J. jaculus*
Jac 27	OL898614	/	*J. hirtipes*
Jac 28	OL898649	MT340280	*J. jaculus*
Jac 29	OL898633	MT340281	*J. jaculus*
Jac 30	OL898635	/	*J. jaculus*
Jac 31	OL898636	MT340283	*J. jaculus*
Jac 32	OL898622	MT340284	*J. hirtipes*
Jac 33	OL898640	MT340285	*J. jaculus*
Jac 34	OL898623	MT340286	*J. hirtipes*
Jac 35	OL898620	MT340287	*J. hirtipes*
Jac 36	OL898637	MT340288	*J. jaculus*
Jac 37	OL898639	MT340289	*J. jaculus*
Jac 38	OL898638	MT340290	*J. jaculus*
Jac 39	OL898618	MT340291	*J. hirtipes*
Jac 40	OL898612	MT340293	*J. hirtipes*
Jac 41	OL898611	MT340294	*J. hirtipes*

Jac 42	/	MT340295	*J. hirtipes*
Jac 43	OL898610	MT340296	*J. hirtipes*
Jac 44	OL898609	MT340297	*J. hirtipes*
Jac 45	OL898630	MT340298	*J. jaculus*
Jac 46	OL898629	MT340299	*J. jaculus*

References

1. Ghawar, W.; Chaouch, M.; Ben Abderrazak, S.; Snoussi, M.A.; Salem, S.; Chouchen, S.; Bouaoun, A.; Ben Salah, A.; Bettaieb, J. Evaluation of the Taxonomic Status of Lesser Egyptian Jerboa, Jaculus jaculus: First Description of New Phylogroups in Tu-nisia. *Animals* **2022**, *12*, 758. https://doi.org/10.3390/ani12060758.

Supplementary Table S2: Accessions number of Tunisian *Leishmania* sequences, their hosts, locations and haplotypes.

Accessions Numbers	Species	Host	Location	Haplotypes (Figure 5)
KY963123	*Leishmania infantum*	*Psammomys obesus*	Sidi Bouzid	Hap 1
KY963124	*Leishmania infantum*	*Psammomys obesus*	Sidi Bouzid	Hap 1
KY963125	*Leishmania infantum*	*Psammomys obesus*	Sidi Bouzid	Hap 1
KY963126	*Leishmania infantum*	*Psammomys obesus*	Sidi Bouzid	Hap 1
KY963127	*Leishmania infantum*	*Psammomys vexilaris*	Kebili	Hap 1
KY963128	*Leishmania infantum*	*Psammomys vexilaris*	Kebili	Hap 1
KY963129	*Leishmania infantum*	*Psammomys vexilaris*	Kebili	Hap 1
MF597933	*Leishmania infantum*	Sand flies	Zagouan	Hap 1
MF597934	*Leishmania infantum*	Sand flies	Zagouan	Hap 1
MF693883	*Leishmania infantum*	Dog	Tunis	Hap 1
MF693885	*Leishmania infantum*	Dog	Tunis	Hap 1
MF693886	*Leishmania infantum*	Dog	Tunis	Hap 1
MF693887	*Leishmania infantum*	Dog	Tunis	Hap 1
MF693888	*Leishmania infantum*	Dog	Tunis	Hap 1
MF693889	*Leishmania infantum*	Dog	Tunis	Hap 1
MH686281	*Leishmania infantum*	Sand flies	Monastir	Hap 1
MH997874	*Leishmania infantum*	Dog	Tunis	Hap 1
MH997875	*Leishmania infantum*	Dog	Tunis	Hap 1
MH997876	*Leishmania infantum*	Dog	Tunis	Hap 1
MK474640	*Leishmania infantum*	Sand flies	Kairouan	Hap 1
MK474641	*Leishmania infantum*	Sand flies	Kairouan	Hap 1
MK474642	*Leishmania infantum*	Human	Kairouan	Hap 1
KY963122	*Leishmania infantum*	*Psammomys obesus*	Sidi Bouzid	Hap 6
MF693884	*Leishmania infantum*	Dog	Tunis	Hap 5
FN677342	*Leishmania major*	Human	Sfax	Hap 2
MK463621	*Leishmania major*	Sand flies	Kairouan	Hap 2
MK463622	*Leishmania major*	Sand flies	Kairouan	Hap 2
MK463623	*Leishmania major*	Sand flies	Kairouan	Hap 2
MK463625	*Leishmania major*	Sand flies	Kairouan	Hap 2
MK463626	*Leishmania major*	Sand flies	Kairouan	Hap 2
MK463627	*Leishmania major*	Sand flies	Kairouan	Hap 2
MK463628	*Leishmania major*	Sand flies	Kairouan	Hap 2
MK463629	*Leishmania major*	Human	Kairouan	Hap 2
MW058081 (jac16)	*Leishmania major*	*Jaculus jaculus*	Tataouine	Hap 2
MW058076 (jac11)	*Leishmania major*	*Jaculus hirtipes*	Tataouine	Hap 2
MW058078 (jac13)	*Leishmania major*	*Jaculus hirtipes*	Tataouine	Hap 2
MW058079 (jac14)	*Leishmania major*	*Jaculus hirtipes*	Tataouine	Hap 2
MW058082 (jac17)	*Leishmania major*	*Jaculus hirtipes*	Tataouine	Hap 2
MW058083 (jac18)	*Leishmania major*	*Jaculus hirtipes*	Tataouine	Hap 2
MW058085 (jac20)	*Leishmania major*	*Jaculus jaculus*	Tataouine	Hap 2
MW058088 (jac35)	*Leishmania major*	*Jaculus hirtipes*	Tataouine	Hap 2
MW058069 (jac4)	*Leishmania major*	*Jaculus hirtipes*	Tataouine	Hap 2

MW058070 (jac5)		*Jaculus hirtipes*	Tataouine	
MW058072 (jac7)		*Jaculus hirtipes*	Tataouine	
MW058073 (jac8)		*Jaculus hirtipes*	Tataouine	
MW058074 (jac9)		*Jaculus hirtipes*	Tataouine	
MW058084 (jac19)		*Jaculus jaculus*	Tataouine	
MW058066 (jac1)		*Jaculus jaculus*	Tataouine	
MW058075 (jac10)		*Jaculus jaculus*	Tataouine	
MW058077 (jac12)		*Jaculus jaculus*	Tataouine	
MW058080 (jac15)		*Jaculus jaculus*	Tataouine	
MW058067 (jac2)		*Jaculus jaculus*	Tataouine	
MW058068 (jac3)		*Jaculus jaculus*	Tataouine	
MW058071 (jac6)		*Jaculus jaculus*	Tataouine	
JN242001		*Ctenodactylus gundi*	Metlaoui	Hap 7
MK463624		Sand flies	Kairouan	Hap 3
AJ300485		Human	Tataouine	Hap 4
JF719995		*Ctenodactylus gundi*	Tataouine	
JF719996		*Ctenodactylus gundi*	Metlaoui	
JN104588		Sand flies	Tataouine	
KY963130		*Psammomys vexilaris*	Kebili	
KY963131		*Psammomys vexilaris*	Kebili	
KY963132		*Psammomys vexilaris*	Kebili	
MK474646	*Leishmania tropica* *(L. killicki)*	Sand flies	Kairouan	
MK474647		Sand flies	Kairouan	
MK474648		Sand flies	Kairouan	
MK474649		Sand flies	Kairouan	
MK474651		Sand flies	Kairouan	
MK474652		Sand flies	Kairouan	
MW058086 (jac34)		*Jaculus hirtipes*	Tataouine	
MW058087 (jac43)		*Jaculus hirtipes*	Tataouine	
MK474653		Human	Kairouan	Hap 8
MK474650		Sand flies	Kairouan	Hap 9

Supplementary Table S3: Accessions number of *Leishmania* sequences detected from rodent all over the world, their hosts, locations and haplotypes.

Accessions Numbers	Species	Host	Location	Haplotypes (Figure 6)
MF977313	*Leishmania infantum*	*Mus musculus*	Morocco	Hap 16
MF977314		*Rattus rattus*	Morocco	
MF977315		*Rattus norvegicus*	Morocco	
JX151015		*Rhombomys opimus*	Iran	
KY963123		*Psammomys obesus*	Tunisia	
KY963124		*Psammomys obesus*	Tunisia	
KY963126		*Psammomys obesus*	Tunisia	
KY963127		*Psammomys vexilaris*	Tunisia	
KY963122		*Psammomys obesus*	Tunisia	Hap 18
KY963125		*Psammomys obesus*	Tunisia	
KY963129		*Psammomys vexilaris*	Tunisia	
KY963128		*Psammomys vexilaris*	Tunisia	Hap 19
JN860730	*Leishmania major*	*Rhombomys opimus*	Iran	Hap 12
JN860731		*Rhombomys opimus*	Iran	
JN860732		*Rhombomys opimus*	Iran	
JN860734		*Rhombomys opimus*	Iran	
JN860735		*Rhombomys opimus*	Iran	
JN860736		*Rhombomys opimus*	Iran	
JN860739		*Rhombomys opimus*	Iran	
JN860740		*Rhombomys opimus*	Iran	
JN860741		*Rhombomys opimus*	Iran	
JN860742		*Rhombomys opimus*	Iran	
JN860745		*Rhombomys opimus*	Iran	
JN860746		*Rhombomys opimus*	Iran	
JN860754		*Rhombomys opimus*	Iran	
JN860757		*Rhombomys opimus*	Iran	
KJ194177		*Meriones libycus*	Iran	
KJ194178		*Meriones libycus*	Iran	
KJ194179		*Meriones libycus*	Iran	
KJ194180		*Meriones libycus*	Iran	
KJ194181		*Meriones libycus*	Iran	
KJ577703		*Rhombomys opimus*	Iran	
KJ577704		*Rhombomys opimus*	Iran	
KJ577705		*Rhombomys opimus*	Iran	
KJ577706		*Rhombomys opimus*	Iran	
KJ577707		*Rhombomys opimus*	Iran	
KJ577708		*Rhombomys opimus*	Iran	
KF152937		*Meriones libycus*	Iran	Hap 17

MW058081 (jac16)		*Jaculus jaculus*	Tunisia	
MW058076 (jac11)		*Jaculus hirtipes*	Tunisia	
MW058078 (jac13)		*Jaculus hirtipes*	Tunisia	
MW058079 (jac14)		*Jaculus hirtipes*	Tunisia	
MW058082 (jac17)		*Jaculus hirtipes*	Tunisia	
MW058083 (jac18)		*Jaculus hirtipes*	Tunisia	
MW058085 (jac20)		*Jaculus jaculus*	Tunisia	
MW058088 (jac35)		*Jaculus hirtipes*	Tunisia	
MW058069 (jac4)		*Jaculus hirtipes*	Tunisia	
MW058070 (jac5)		*Jaculus hirtipes*	Tunisia	
MW058072 (jac7)		*Jaculus hirtipes*	Tunisia	
MW058073 (jac8)		*Jaculus hirtipes*	Tunisia	
MW058074 (jac9)		*Jaculus hirtipes*	Tunisia	
MW058084 (jac19)		*Jaculus jaculus*	Tunisia	
MW058066 (jac1)		*Jaculus jaculus*	Tunisia	
MW058075 (jac10)		*Jaculus jaculus*	Tunisia	
MW058077 (jac12)		*Jaculus jaculus*	Tunisia	
MW058080 (jac15)		*Jaculus jaculus*	Tunisia	
MW058067 (jac2)		*Jaculus jaculus*	Tunisia	
MW058068 (jac3)		*Jaculus jaculus*	Tunisia	
MW058071 (jac6)		*Jaculus jaculus*	Tunisia	
JN242001		*Ctenodactylus gundi*	Tunisia	Hap 11
JN860737		*Rhombomys opimus*	Iran	Hap 13
JN860738		*Rhombomys opimus*	Iran	Hap 14
JN860750		*Rhombomys opimus*	Iran	Hap 15
JF719995	*Leishmania tropica (L. killicki)*	*Ctenodactylus gundi*	Tunisia	Hap 10
JF719996		*Ctenodactylus gundi*	Tunisia	
KY963130		*Psammomys vexilaris*	Tunisia	
KY963131		*Psammomys vexilaris*	Tunisia	
KY963132		*Psammomys vexilaris*	Tunisia	
MW058086 (jac34)		*Jaculus hirtipes*	Tunisia	
MW058087 (jac43)		*Jaculus hirtipes*	Tunisia	
MF977312		*Mus musculus*	Morocco	Hap 20
